# Functional Independence of Taiwanese Children with Silver–Russell Syndrome

**DOI:** 10.3390/diagnostics15091109

**Published:** 2025-04-27

**Authors:** Hung-Hsiang Fang, Chung-Lin Lee, Chih-Kuang Chuang, Huei-Ching Chiu, Ya-Hui Chang, Yuan-Rong Tu, Yun-Ting Lo, Jun-Yi Wu, Yen-Yin Chou, Chung-Hsing Wang, Shio-Jean Lin, Shao-Yin Chu, Chen Yang, Tsung-Ying Ou, Hsiang-Yu Lin, Shuan-Pei Lin

**Affiliations:** 1 Department of Pediatrics, MacKay Memorial Hospital, Taipei 104, Taiwan; spty871029@hotmail.com (H.-H.F.); clampcage@gmail.com (C.-L.L.); g880a01@mmh.org.tw (H.-C.C.); wish1001026@gmail.com (Y.-H.C.); 2 Department of Pediatrics, Tri-Service General Hospital, National Defense Medical Center, Taipei 114, Taiwan; 3 Institute of Clinical Medicine, National Yang-Ming Chiao-Tung University, Taipei 112, Taiwan; 4 International Rare Disease Center, MacKay Memorial Hospital, Taipei 104, Taiwan; andy11tw.e347@mmh.org.tw (Y.-T.L.); wl01723138@gmail.com (J.-Y.W.); 5 Department of Medicine, MacKay Medical College, New Taipei City 252, Taiwan; 6 MacKay Junior College of Medicine, Nursing and Management, Taipei 112, Taiwan; 7 Department of Medical Research, MacKay Memorial Hospital, Taipei 104, Taiwan; mmhcck@gmail.com (C.-K.C.); likemaruko@hotmail.com (Y.-R.T.); 8 College of Medicine, Fu-Jen Catholic University, Taipei 242, Taiwan; 9 Department of Pediatrics, National Cheng Kung University Hospital, College of Medicine, National Cheng Kung University, Tainan 704, Taiwan; yenyin@mail.ncku.edu.tw; 10 Division of Medical Genetics, Pediatric Endocrinology and Metabolism, China Medical University Children’s Hospital, China Medical University, Taichung 404, Taiwan; a22340961@yahoo.com.tw; 11 Department of Pediatrics, Genetic Counseling Center, Chi Mei Medical Center, Tainan 710, Taiwan; shiojean@gmail.com; 12 Department of Pediatrics, Buddhist Tzu-Chi General Hospital, Hualien 970, Taiwan; hushaoyin@gmail.com; 13 Department of Pediatrics, Taipei Medical University Hospital, Taipei 110, Taiwan; yeungsann@yahoo.com.tw; 14 Department of Pediatrics, Dalin Tzu Chi Hospital, Buddhist Tzu Chi Medical Foundation, Chiayi 622, Taiwan; sleep12358@gmail.com; 15 Department of Medical Research, China Medical University Hospital, China Medical University, Taichung 404, Taiwan; 16 Department of Infant and Child Care, National Taipei University of Nursing and Health Sciences, Taipei 112, Taiwan

**Keywords:** independent living, Silver–Russell syndrome, Taiwan, WeeFIM

## Abstract

**Background:** Silver–Russell syndrome (SRS) is a genetic disorder characterized by prenatal and postnatal growth retardation. Affected individuals commonly present with low birth weight, intrauterine growth restriction, postnatal short stature, hemihypotrophy, characteristic facial features, and body asymmetry. **Methods:** This study includes 24 Taiwanese children with SRS aged 2 years to 13 years and 3 months who were recruited at MacKay Memorial Hospital and other Taiwan hospitals between January 2013 and December 2024. Functional independence was assessed using the Functional Independence Measure for Children (WeeFIM) to evaluate self-care, mobility, and cognition domains. **Results:** The mean total WeeFIM score was 106.9 ± 23.2 (range: 54–126), with mean self-care, mobility, and cognition scores of 44.4 ± 13.8 (maximum 56), 32.4 ± 5.1 (maximum 35), and 30.2 ± 6.0 (maximum 35), respectively. The results of the restricted cubic spline analysis reveal a clear positive linear correlation before school age (approximately 72 months), followed by a plateau (*p* for nonlinearity < 0.05). Traceable molecular data were available for thirteen participants, of whom nine (69%) had loss of methylation at chromosome 11p15 (11p15LOM), and four (31%) had maternal uniparental disomy of chromosome 7 (upd(7)mat). Of the 24 children, 46% required assistance with bathing, which was strongly correlated with self-care ability and body height. In contrast, most of the children had independence in mobility tasks such as walking and stair climbing. However, some required support in cognitive tasks, including problem-solving, comprehension, and expression. Overall, the included children reached a functional plateau later than the normative population, with the greatest delays in self-care and mobility domains. **Conclusions:** This study highlights that Taiwanese children with SRS require support in self-care and cognitive tasks. Functional independence in self-care and mobility domains was positively associated with body height. The WeeFIM questionnaire effectively identified strengths and limitations, emphasizing the need for individualized support in daily activities.

## 1. Introduction

Silver–Russell syndrome (SRS; OMIM #180860) is a rare imprinting disorder characterized by prenatal and postnatal growth retardation [1,2]. The condition was first described by Silver et al. in 1953 [3] and Russell et al. in 1954 [4], who reported children with low birth weight, intrauterine growth restriction, postnatal short stature, hemihypotrophy, characteristic facial features, and body asymmetry. The estimated incidence of SRS ranges from 1:30,000 to 1:100,000, and nearly all individuals with SRS are born small for their gestational age [5].

The clinical diagnosis of SRS currently relies on the Netchine–Harbison clinical scoring system, which has been shown to have high sensitivity and strong negative predictive value [5,6,7,8,9]. The Netchine–Harbison system includes six criteria: small for gestational age (birth weight and/or birth length), postnatal growth failure, relative macrocephaly at birth, protruding forehead, body asymmetry, and feeding difficulties and/or low body mass index. A diagnosis of SRS can also be established through molecular testing, with the most common genetic findings including loss of methylation at chromosome 11p15 (11p15LOM) and maternal uniparental disomy of chromosome 7 (upd(7)mat) [10,11]. In rare cases, copy number variants and monogenic pathogenic variants in imprinted (*CDKN1C*, *IGF2*) and non-imprinted (*PLAG1*, *HMGA2*) genes have been demonstrated to contribute to the etiology [2,10,12,13].

As growth retardation can result from genetic, maternal, or environmental factors, comprehensive phenotypic profiling and timely molecular analysis are essential to diagnose SRS. Patients with imprinting center 1 hypomethylation are more likely to exhibit classical SRS features such as asymmetry, fifth-finger clinodactyly, and congenital anomalies compared to those with upd(7)mat [1,12].

The Functional Independence Measure for Children (WeeFIM) questionnaire is a practical tool for assessing functional outcomes [14,15], and it has been adapted for use in Chinese children [16]. Recognizing the need to assess the impact of SRS on functional independence, this study aims to quantify functional performance in Taiwanese children with SRS using the WeeFIM questionnaire, identify associated factors, and characterize functional limitations and impacts on daily caregiving.

## 2. Methods

### 2.1. Study Population

Twenty-four children with SRS aged from 2 years to 13 years and 3 months and their parents were recruited at MacKay Memorial Hospital and other Taiwan hospitals between January 2013 and December 2024. The parents and children completed the WeeFIM questionnaire at the clinic. The WeeFIM questionnaire was completed jointly by the child and their parent or legal guardian, with guidance and clarification provided by clinicians during outpatient clinic visits. This study was approved by the Institutional Review Board of MacKay Memorial Hospital (Reference number: 21MMHIS109e, approval date: 1 October 2021). All participants provided assent, while their parents or legal guardians signed a parental consent form.

Patient profiles and medical interventions were documented, and clinical features including molecular type, body height, early intervention history, and age at questionnaire completion were recorded. The diagnosis of SRS was confirmed either through molecular testing or clinical assessment. Early intervention history was obtained from parental reports or medical records. For patients with multiple questionnaire records, only the first completed questionnaire was selected.

### 2.2. WeeFIM Questionnaire

The WeeFIM questionnaire was designed for primary caregivers to directly assess their child’s functional abilities and developmental disabilities [14,17]. The Chinese version of the WeeFIM questionnaire was used in this study to assess the functional independence of the enrolled children [16,18]. It was designed for children aged from 6 months to 7 years and can be used for individuals up to 21 years of age with developmental disabilities [19,20,21,22,23].

The WeeFIM questionnaire consists of 18 items categorized into three functional domains: self-care, mobility, and cognition. The self-care domain includes eight items: eating, grooming, bathing, upper-body dressing, lower-body dressing, toileting, bladder management, and bowel management. The mobility domain includes five items: chair transfer, toilet transfer, tub transfer, walking, and stair climbing. The cognition domain consists of five items: comprehension, expression, social interaction, problem-solving, and memory.

Each item is rated on a seven-point ordinal scale that reflects the level of assistance required for task completion, with higher scores corresponding to greater functional independence. A score of 1 indicates total assistance, where the participant is able to perform less than 25% of the task, while a score of 2 represents maximal assistance, with the participant able to complete 25–49% of the task. Moderate assistance (score of 3) indicates that the participant can perform 50–74% of the task, and minimal assistance (score of 4) denotes that the participant can perform at least 75% of the task. A score of 5 indicates that supervision, setup, or standby assistance is required, while a score of 6 represents modified independence, meaning that the participant can complete the task with an assistive device or with some safety or efficiency concerns. A score of 7 represents complete independence, indicating that the participant can complete the task safely and timely without the need of assistance or assistive devices [24].

The WeeFIM questionnaire has been widely used to assess functional abilities in children with developmental disorders, and it provides a standardized measure of self-care, mobility, and cognitive functioning. Scores ranging from 1 to 5 indicate dependence, requiring assistance for daily activities, whereas scores of 6 and 7 signify independence with no external support. The self-care, mobility, and cognition domain scores range from 8 to 56, 5 to 35, and 5 to 35, respectively, with a total WeeFIM score ranging from 18 to 126 [25].

### 2.3. Statistical Analysis

Descriptive statistics were used, and the results are presented as the median (interquartile range, IQR) and mean (standard deviation, SD), unless otherwise stated. All participants were under 16 years old. Due to the limited age range, the 24 enrolled children were stratified into three age groups (0–5, 6–10, and 11–15 years) for functional performance evaluation. The patients’ WeeFIM scores were compared to normative Chinese children [16]. The differences in continuous variables (e.g., age, height, WeeFIM scores) among groups were analyzed using one-way analysis of variance (ANOVA), with Bonferroni correction applied for pairwise comparisons. Consistent with similar studies [26,27], the relationship between age, height, and WeeFIM scores was analyzed using linear regression, with age and height modeled as restricted cubic splines (RCS) with knots placed at the 10th, 50th, and 95th percentiles. RCS modeling was performed using R software, version 4.4.3 (R Foundation for Statistical Computing, Vienna, Austria), and the “rms” package version 7.0–0 (Frank E. Harrell Jr). All other statistical analyses were conducted using IBM SPSS Statistics software version 25.0 (IBM Corp., Armonk, NY, USA). A 2-sided *p* value of <0.05 was considered statistically significant.

## 3. Results

A total of 24 children (12 male and 12 female) with SRS were included in this study. Their age ranged from 2 years to 13 years and 3 months, with a median age at enrollment of 5 years and 8 months. The diagnosis of SRS was confirmed either by molecular studies or using clinical assessment [5]. Traceable molecular data were available for thirteen participants, of whom nine (69%) had 11p15LOM, and four (31%) had upd(7)mat. Data on height were available for 16 participants, and 8 children had a history of receiving early intervention.

**Figure 1:**
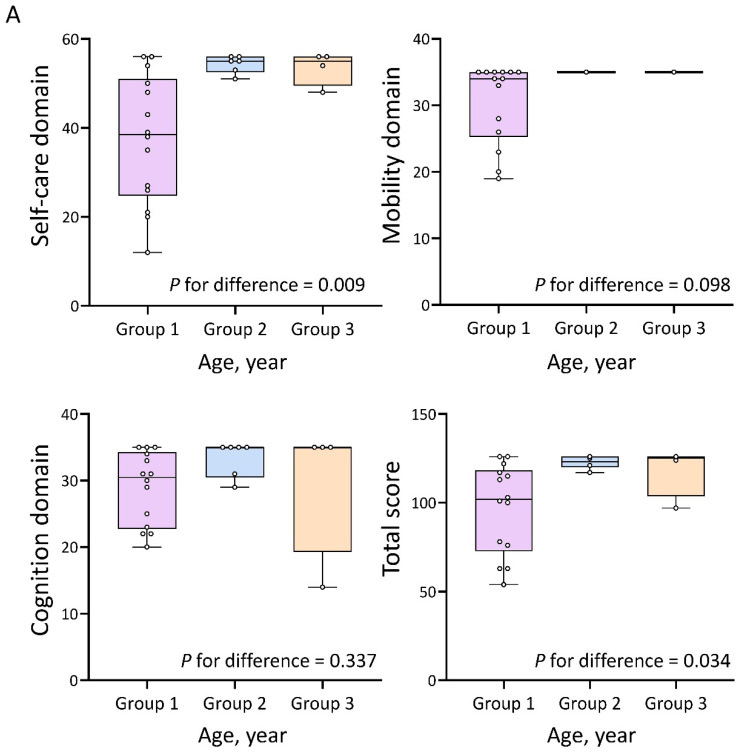

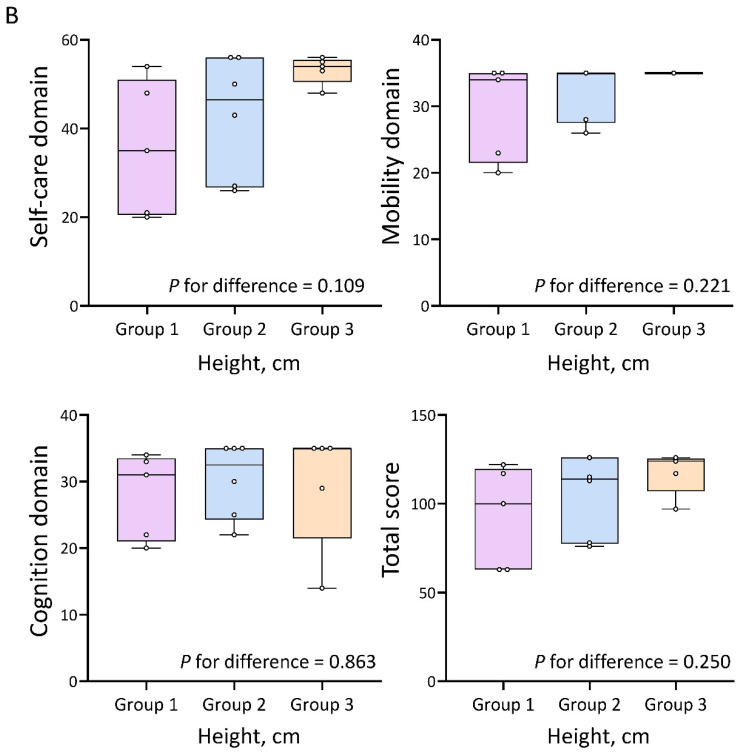
(**A**) Box plot illustrating the median and quartiles of total WeeFIM scores and domain-specific scores across three age groups: 2.0–5.9 yrs, 6.0–10.9 yrs, and 11.0–15.9 yrs. (**B**) Box plot illustrating the median and quartiles of total WeeFIM scores and domain-specific scores across three height-based groups: 71.6–88.0 cm, 88.1–120.3 cm, and 120.4–135.5 cm.

The total WeeFIM score of the enrolled children ranged from 54 to 126 (median 117). Table 1 summarizes the total, mean, median, and IQR scores for each domain in the three age groups. The mean total WeeFIM score in the overall cohort was 106.9 ± 23.2 (range: 54–126), and the mean self-care, mobility, and cognition scores were 44.4 ± 13.8 (maximum 56), 32.4 ± 5.1 (maximum 35), and 30.2 ± 6.0 (maximum 35), respectively. The median IQR scores for the self-care, mobility, and cognition domains were 50.5 (36.5–55.5), 35.0 (33.5–35.0), and 32.0 (27.0–35.0), respectively. When grouped by age, significant differences were observed between groups in the self-care domain and total WeeFIM scores. The box plot in Figure 1A illustrates the distribution of scores across the self-care, mobility, and cognition domains, as well as total WeeFIM scores. The 16 children with recorded height data were divided into three groups based on height, from shortest to tallest. However, no significant differences were found between the height groups in self-care, mobility, or cognition domains, or in total WeeFIM score (Table 2). Figure 1B presents a box plot illustrating the distribution of self-care, mobility, cognition, and total WeeFIM scores among the participants grouped by height.

**Figure 2:**
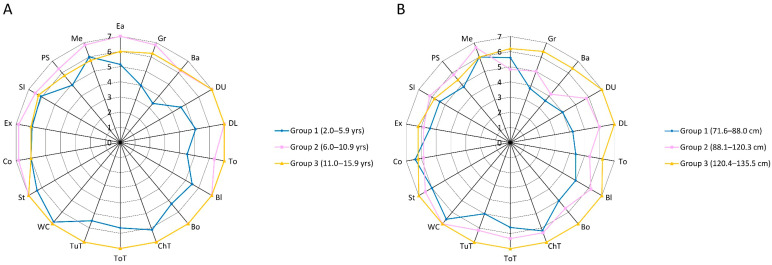
(**A**) WeeFIM profiles of the study participants stratified by three age groups; (**B**) WeeFIM profiles of the study participants stratified by three height-based groups. Ba = bathing; Bl = bladder; Bo = bowel; ChT = bed/chair/wheelchair transfer; Co = comprehension; DL = dressing (lower); DU = dressing (upper); Ea = eating; Ex = expression; Gr = grooming; Me = memory; PS = problem-solving; SI = social interaction; St = stairs; To = toileting; ToT = toilet transfer; TuT = tub/shower transfer; WC = walk/wheelchair.

Based on the WeeFIM profiles of the participants stratified by age and height (Figure 2A,B), the lowest performance was in the bathing task. Table 3 summarizes the WeeFIM scores for the children requiring assistance or supervision versus those who were independent across the three domains. In the self-care domain, from 17% to 46% of the participants had scores ranging from 1 to 4, indicating varying levels of assistance required for different self-care tasks. Notably, 46% of the participants needed assistance with bathing. In contrast, most children demonstrated independence in mobility tasks, with 96% walking independently and 92% able to climb stairs without assistance. Despite their mobility independence, some children required support in problem-solving (33%), comprehension (21%), and expression (21%), highlighting cognitive challenges in daily functioning. To compare the functional development of the included children with the general population, Table 4 presents the age at which the 50th percentile of the included children attained level 6 on the WeeFIM scale. The attainment order differed slightly from the normative functional independence profile for Chinese children [16].

**Figure 3:**
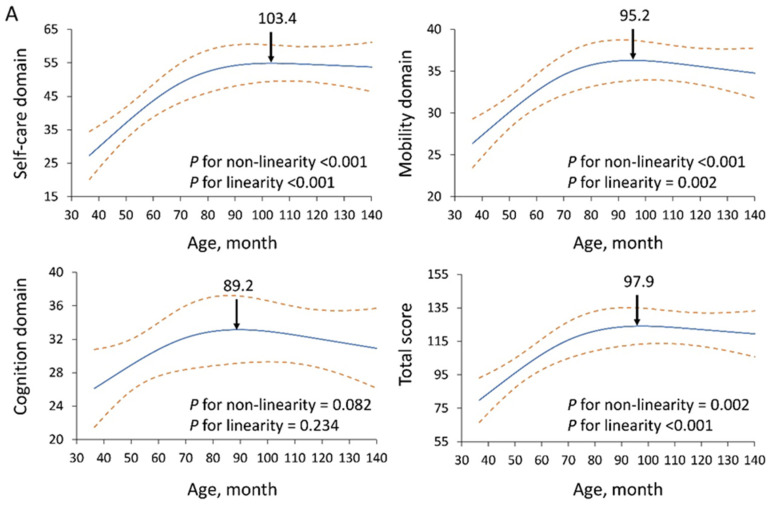

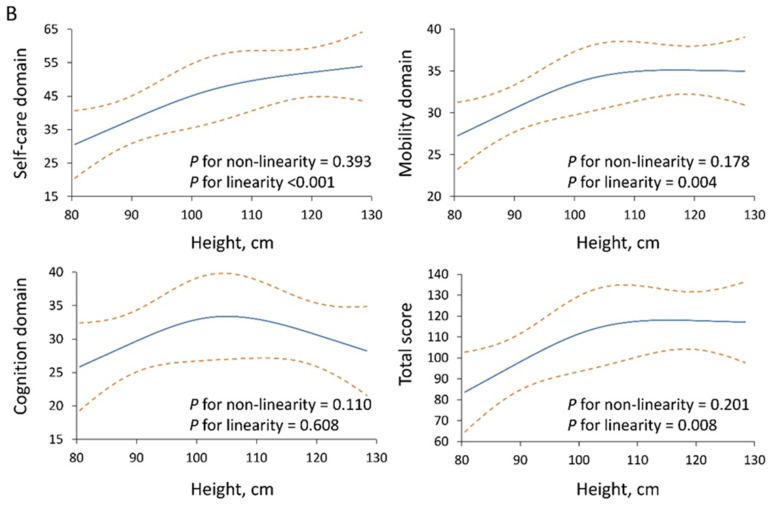
(**A**) Linear and nonlinear relationships of age-related trends in self-care, mobility, cognition, and total WeeFIM scores. (**B**) Linear and nonlinear relationships of height-related trends in self-care, mobility, cognition, and total WeeFIM scores. The solid blue line represents the predicted mean score based on a restricted cubic spline regression model and the dashed orange lines represent the 95% confidence intervals of the predicted scores.

We utilize restricted cubic splines in Figure 3 to visualize the relationships between age, height, and WeeFIM scores. The results show that the relationship between age and WeeFIM scores was generally nonlinear, except for the cognition domain, where the nonlinearity significance was 0.082. A clear positive linear correlation was observed before school age (approximately 72 months), after which the scores plateaued at varying ages depending on the specific WeeFIM domains (Figure 3A). By contrast, the relationship between height and WeeFIM scores was generally linear, except for the cognition domain (*p* for linearity = 0.608) (Figure 3B).

The results also show no significant differences in the mean total WeeFIM score between those with the 11p15LOM (105.3 ± 25.5) and upd(7)mat (109.3 ± 11.3) type and the corresponding self-care (42.9 ± 14.4 and 47.8 ± 6.3), mobility (31.6 ± 5.6 and 34.5 ± 1.0), and cognition (30.9 ± 6.0 and 27.0 ± 8.8) scores. Sex-specific WeeFIM scores have not previously been reported, and our results show no significant differences in the total score between boys (103.9 ± 21.7) and girls (109.9 ± 25.2) and the corresponding self-care (42.3 ± 12.5 and 46.4 ± 15.2), mobility (32.4 ± 4.3 and 32.3 ± 6.0), and cognition (29.2 ± 7.3 and 31.2 ± 4.6) scores.

## 4. Discussion

In this study, we used the WeeFIM questionnaire to assess disabilities in self-care, mobility, and cognition domains and characterize the range of functional performance across these domains in children with SRS aged from 2 years to 13 years and 3 months. Overall, 79% of the children were independent in mobility, compared with 58% in cognition and 54% in self-care. These results are consistent with a previous study [28]. Among the children across the different age and height groups, the lowest WeeFIM subscores were observed in bathing (Figure 2A,B), and bathing performance was strongly correlated with self-care ability and body height.

The WeeFIM total score, self-care, and mobility domains were positively correlated with age (*p* < 0.05). Among the age groups, significant differences were observed in the self-care and total WeeFIM scores. The increasing trends plateaued at 103.4 months (normative population: 72 months) in the self-care domain, 95.2 months (54 months) in the mobility domain, 89.2 months (80 months) in the cognition domain, and 97.9 months (72 months) in the total WeeFIM score (Figure 3A). The children with SRS reached a functional plateau later than the normative population [16] in all three domains and the total WeeFIM score, with the most pronounced delays observed in the self-care and mobility domains.

RCS regression was performed to explore nonlinearity, followed by a two-piecewise linear regression model to determine turning points. The RCS analysis indicated nonlinear relationships between age and self-care (*p* for nonlinearity < 0.001; Figure 3A), mobility (*p* for nonlinearity < 0.001; Figure 3A), and total WeeFIM scores (*p* for nonlinearity = 0.002; Figure 3A). However, the relationship between age and cognition scores was linear (*p* for nonlinearity = 0.082; Figure 3A). To further validate these findings, simple linear regression was performed. Significant linear associations were observed in the self-care, mobility, and total WeeFIM scores (*p* < 0.05), whereas the cognition domain did not reach statistical significance (*p* = 0.234). Overall, there were progressive increases in self-care, mobility, cognition, and total WeeFIM scores with age, which plateaued upon reaching functional maturity. RCS analysis did not indicate a nonlinear relationship between height and self-care (*p* = 0.393; Figure 3B), mobility (*p* = 0.178; Figure 3B), cognition (*p* = 0.110; Figure 3B), or total WeeFIM score (*p* = 0.201; Figure 3B). To further assess linearity, simple linear regression was performed. Significant linear associations were identified in self-care, mobility, and total WeeFIM scores (*p* < 0.05), whereas the cognition domain did not reach significance (*p* = 0.608). Functional scores showed a positive association with height, except in the cognition domain. The children with SRS followed a different sequence in attaining functional performance, and specific tasks such as bathing and upper-body dressing took longer to achieve level 6 compared to the general population.

This study has several limitations. Firstly, due to the rarity of SRS, the sample size was small, which may limit the generalizability of the findings. Secondly, the participants were relatively young, potentially affecting the assessment of functional milestones in older individuals. In addition, as the original version of the WeeFIM questionnaire was not developed in Chinese, the translation may have introduced subtle nuances that were not fully recognized. To ensure accuracy and cultural relevance, further validation of the translated version is necessary. Thirdly, the records of the children were missing some data on height, molecular type, early intervention history, and developmental delays. To better understand the impact of these factors on motor and cognitive functioning in children with SRS, larger and more comprehensive studies are required.

## 5. Conclusions

The WeeFIM questionnaire responses reveal that the children with SRS had greater independence in mobility compared to self-care and cognitive functions. Our findings highlight that Taiwanese children with SRS require additional support in self-care and cognitive tasks. We also found that functional independence in self-care and mobility domains was positively associated with body height. These findings provide valuable insights for clinicians in identifying the functional strengths and challenges of children with SRS, facilitating the development of individualized support strategies to enhance daily living and overall quality of life.

## Figures and Tables

**Table 1:** WeeFIM scores of the children with Silver–Russell syndrome grouped by age.

Variable	Total (2.0–15.9 Years)	Group 1 (2.0–5.9 Years)	Group 2 (6.0–10.9 Years)	Group 3 (11.0–15.9 Years)	*p* Value
Number of children	24	14	6	4	
Age, year					
Mean ± standard deviation	6.7 ± 3.3	4.3 ± 1.2	8.6 ± 1.0 ^a^	12.3 ± 0.7 ^ab^	<0.001
Range	2.0–13.3	2.0–5.9	7.3–9.6	11.9–13.3	
Self-care score					0.009
Mean ± standard deviation	44.4 ± 13.8	37.5 ± 14.5	54.3 ± 2.0 ^a^	53.5 ± 3.8	
Median [25th percentile, 75th percentiles]	50.5 [36.5, 55.5]	38.5 [26.0, 50.0]	55.0 [53.0, 56.0]	55.0 [51.0, 56.0]	
Mobility score					0.098
Mean ± standard deviation	32.4 ± 5.1	30.5 ± 6.1	35.0 ± 0	35.0 ± 0	
Median [25th percentile, 75th percentiles]	35.0 [33.5, 35.0]	34.0 [26.0, 35.0]	35.0 [35.0, 35.0]	35.0 [35.0, 35.0]	
Cognition score					0.337
Mean ± standard deviation	30.2 ± 6.0	28.9 ± 5.5	33.3 ± 2.7	29.8 ± 10.5	
Median [25th percentile, 75th percentiles]	32.0 [27.0, 35.0]	30.5 [23.0, 34.0]	35.0 [31.0, 35.0]	35.0 [24.5, 35.0]	
Total score					0.034
Mean ± standard deviation	106.9 ± 23.2	96.9 ± 25.3	122.7 ± 3.6	118.3 ± 14.2	
Median [25th percentile, 75th percentiles]	117.0 [98.5, 125.5]	102.0 [76.0, 117.0]	123.0 [121.0, 126.0]	125.0 [110.5, 126.0]	

Abbreviation: WeeFIM, functional independence measure for children; “a” and “b” denote significant differences compared to the 2.0–5.9 years and 6.0–10.9 years age groups, respectively, following Bonferroni correction.

**Table 2:** WeeFIM scores of children with Silver–Russell syndrome grouped by height.

Variable	Total (71.6–135.5 cm)	Group 1 (71.6–88.0 cm)	Group 2 (88.1–120.3 cm)	Group 3 (120.4–135.5 cm)	*p* Value
Number of children	16	5	6	5	
Height, cm					
Mean ± standard deviation	103.9 ± 19.2	83.0 ± 6.7	103.0 ± 11.0 ^a^	125.8 ± 5.8 ^ab^	<0.001
Range	71.6–135.5	71.6–88.0	88.9–120.3	121.3–135.5	
Self-care score					0.109
Mean ± standard deviation	44.0 ± 13.0	36.0 ± 15.0	43.0 ± 14.0	53.0 ± 3.0	
Median [25th percentile, 75th percentiles]	49.0 [31.0, 55.0]	35.0 [21.0, 48.0]	47.0 [27.0, 56.0]	54.0 [53.0, 55.0]	
Mobility score					0.221
Mean ± standard deviation	32.0 ± 5.0	29.0 ± 7.0	32.0 ± 4.0	35.0 ± 0	
Median [25th percentile, 75th percentiles]	35.0 [31.0, 35.0]	34.0 [23.0, 35.0]	35.0 [28.0, 35.0]	35.0 [35.0, 35.0]	
Cognition score					0.863
Mean ± standard deviation	29.0 ± 7.0	28.0 ± 7.0	30.0 ± 6.0	30.0 ± 9.0	
Median [25th percentile, 75th percentiles]	32.0 [24.0, 35.0]	31.0 [22.0, 33.0]	33.0 [25.0, 35.0]	35.0 [29.0, 35.0]	
Total score					0.250
Mean ± standard deviation	106.0 ± 23.0	93.0 ± 29.0	106.0 ± 23.0	118.0 ± 12.0	
Median [25th percentile, 75th percentiles]	116.0 [88.0, 125.0]	100.0 [63.0, 117.0]	114.0 [78.0, 126.0]	124.0 [117.0, 125.0]	

Abbreviation: WeeFIM, functional independence measure for children; “a” and “b” denote significant differences compared to the 71.6–88.0 cm and 88.1–120.3 cm height groups, respectively, following Bonferroni correction.

**Table 3:** Scores of individual WeeFIM tasks grouped into help, supervision, and no help categories in the children with Silver–Russell syndrome.

Task	Requiring Help (1–4 Points)		Requiring Supervision (5 Points)		Requiring No Help (6–7 Points)	
	*n*	%	*n*	%	*n*	%
Self-care						
Eating	5	21	5	21	14	58
Grooming	9	38	2	8	13	54
Bathing	11	46	0	0	13	54
Dressing upper	8	33	1	4	15	63
Dressing lower	6	25	2	8	16	67
Toileting	8	33	0	0	16	67
Bladder	4	17	1	4	19	79
Bowel	5	21	0	0	19	79
Mobility						
Chair transfer	1	4	4	17	19	79
Toilet transfer	2	8	3	13	19	79
Tub transfer	4	17	1	4	19	79
Walking	0	0	1	4	23	96
Stairs	2	8	0	0	22	92
Cognition						
Comprehension	5	21	0	0	19	79
Expression	5	21	0	0	19	79
Social interaction	3	13	4	17	17	71
Problem-solving	8	33	2	8	14	58
Memory	3	13	2	8	19	79

Abbreviation: WeeFIM, functional independence measure for children.

**Table 4:** Order of 50th percentiles for attaining level 6 for children with Silver–Russell syndrome.

Order of Achievement	Items	25th Percentile (Months)	50th Percentile (Months)	75th Percentile (Months)
1	Walking	48.6	70.0	113.9
2	Stairs	52.2	70.2	113.9
3	Comprehension	52.2	70.5	113.9
4	Expression	52.2	70.5	113.9
5	Social interaction	52.2	70.5	108.5
6	Memory	52.2	70.5	113.9
7	Bladder	60.0	87.5	115.6
8	Bowel	60.0	87.5	115.6
9	Chair transfer	60.0	87.5	115.6
10	Toilet transfer	60.0	87.5	115.6
11	Tub transfer	60.0	87.5	115.6
12	Eating	63.1	88.7	113.9
13	Toileting	61.6	88.7	128.1
14	Problem-solving	60.0	88.7	113.9
15	Dressing lower	66.1	96.0	129.0
16	Grooming	70.0	102.0	115.6
17	Bathing	63.1	102.0	115.6
18	Dressing upper	63.1	102.0	142.3

## Data Availability

All data are present within the article.

## Abbreviations

SRS: Silver–Russell syndrome; 11p15LOM: loss of methylation at chromosome 11p15; upd(7)mat: maternal uniparental disomy of chromosome 7; WeeFIM: Functional Independence Measure for Children; IQR: interquartile range; SD: standard deviation; RCS: restricted cubic spline.

## References

[1] Binder G., Begemann M., Eggermann T., Kannenberg K. (2011). Silver-Russell syndrome. Best. Pract. Res. Clin. Endocrinol. Metab..

[2] Saal H.M., Harbison M.D., Netchine I., Adam M.P., Feldman J., Mirzaa G.M., Pagon R.A., Wallace S.E., Amemiya A. (1993). Silver-Russell Syndrome. GeneReviews^®^.

[3] Silver H.K., Kiyasu W., George J., Deamer W.C. (1953). Syndrome of congenital hemihypertrophy, shortness of stature, and elevated urinary gonadotropins. Pediatrics.

[4] Russell A. (1954). A syndrome of intra-uterine dwarfism recognizable at birth with cranio-facial dysostosis, disproportionately short arms, and other anomalies (5 examples). Proc. R. Soc. Med..

[5] Wakeling E.L., Brioude F., Lokulo-Sodipe O., O’Connell S.M., Salem J., Bliek J., Canton A.P.M., Chrzanowska K.H., Davies J.H., Dias R.P. (2017). Diagnosis and management of Silver-Russell syndrome: First international consensus statement. Nat. Rev. Endocrinol..

[6] Azzi S., Salem J., Thibaud N., Chantot-Bastaraud S., Lieber E., Netchine I., Harbison M.D. (2015). A prospective study validating a clinical scoring system and demonstrating phenotypical-genotypical correlations in Silver-Russell syndrome. J. Med. Genet..

[7] Netchine I., Rossignol S., Dufourg M.-N., Azzi S., Rousseau A., Perin L., Houang M., Steunou V., Esteva B., Thibaud N. (2007). 11p15 imprinting center region 1 loss of methylation is a common and specific cause of typical Russell-Silver syndrome: Clinical scoring system and epigenetic-phenotypic correlations. J. Clin. Endocrinol. Metab..

[8] Dias R.P., Nightingale P., Hardy C., Kirby G., Tee L., Price S., MacDonald F., Barrett T.G., Maher E.R. (2013). Comparison of the clinical scoring systems in Silver-Russell syndrome and development of modified diagnostic criteria to guide molecular genetic testing. J. Med. Genet..

[9] Lin H.-Y., Lee C.-L., Tu Y.-R., Chang Y.-H., Niu D.-M., Chang C.-Y., Chiu P.C., Chou Y.-Y., Hsiao H.-P., Tsai M.-C. (2024). Quantitative DNA Methylation Analysis and Epigenotype-Phenotype Correlations in Taiwanese Patients with Silver-Russell Syndrome. Int. J. Med. Sci..

[10] Kurup U., Lim D.B.N., Palau H., Maharaj A.V., Ishida M., Davies J.H., Storr H.L. (2024). Approach to the Patient With Suspected Silver-Russell Syndrome. J. Clin. Endocrinol. Metab..

[11] Schonherr N., Meyer E., Eggermann K., Ranke M.B., Wollmann H.A., Eggermann T. (2006). (Epi)mutations in 11p15 significantly contribute to Silver-Russell syndrome: But are they generally involved in growth retardation?. Eur. J. Med. Genet..

[12] Lin H.Y., Lee C.L., Fran S., Tu R.Y., Chang Y.H., Niu D.M., Chang C.Y., Chiu P.C., Chou Y.Y., Hsiao H.P. (2021). Epigenotype, Genotype, and Phenotype Analysis of Taiwanese Patients with Silver-Russell Syndrome. J. Pers. Med..

[13] Singh A., Pajni K., Panigrahi I., Khetarpal P. (2023). Clinical and Molecular Heterogeneity of Silver-Russell Syndrome and Therapeutic Challenges: A Systematic Review. Curr. Pediatr. Rev..

[14] Ottenbacher K.J., Msall M.E., Lyon N., Duffy L.C., Granger C.V., Braun S. (1999). Measuring developmental and functional status in children with disabilities. Dev. Med. Child. Neurol..

[15] Liu M., Toikawa H., Seki M., Domen K., Chino N. (1998). Functional Independence Measure for Children (WeeFIM): A preliminary study in nondisabled Japanese children. Am. J. Phys. Med. Rehabil..

[16] Wong V., Wong S., Chan K., Wong W. (2002). Functional Independence Measure (WeeFIM) for Chinese children: Hong Kong Cohort. Pediatrics.

[17] Ottenbacher K.J., Msall M.E., Lyon N., Duffy L.C., Ziviani J., Granger C.V., Braun S., Feidler R.C. (2000). The WeeFIM instrument: Its utility in detecting change in children with developmental disabilities. Arch. Phys. Med. Rehabil..

[18] Wong S.S., Wong V.C. (2007). Functional Independence Measure for Children: A comparison of Chinese and Japanese children. Neurorehabil Neural Repair..

[19] Wong V., Chung B., Hui S., Fong A., Lau C., Law B., Lo K., Shum T., Wong R. (2004). Cerebral palsy: Correlation of risk factors and functional performance using the Functional Independence Measure for Children (WeeFIM). J. Child. Neurol..

[20] Lee C., Lin H., Chuang C., Chiu H., Tu R., Huang Y., Hwu W., Tsai F., Chiu P., Niu D. (2019). Functional independence of Taiwanese patients with mucopolysaccharidoses. Mol. Genet. Genom. Med..

[21] Lee C., Lin H., Tsai L., Chiu H., Tu R., Huang Y., Chien Y., Lee N., Niu D., Chao M. (2018). Functional independence of Taiwanese children with Prader-Willi syndrome. Am. J. Med. Genet. A.

[22] Lin H., Chuang C., Chen Y., Tu R., Chen M., Niu D., Lin S. (2016). Functional independence of Taiwanese children with Down syndrome. Dev. Med. Child. Neurol..

[23] Syu Y.-M., Lee C.-L., Chuang C.-K., Chiu H.-C., Chang Y.-H., Lin H.-Y., Lin S.-P. (2022). Functional Independence of Taiwanese Children with Osteogenesis Imperfecta. J. Pers. Med..

[24] Sperle P.A., Ottenbacher K.J., Braun S.L., Lane S.J., Nochajski S. (1997). Equivalence reliability of the functional independence measure for children (WeeFIM) administration methods. Am. J. Occup. Ther..

[25] Lin H.-Y., Lin S.-P., Lin H.-Y., Hsu C.-H., Chang J.-H., Kao H.-A., Hung H.-Y., Peng C.-C., Lee H.-C., Chen M.-R. (2012). Functional independence of Taiwanese children with VACTERL association. Am J Med Genet A.

[26] Chen Q., Hu P., Hou X., Sun Y., Jiao M., Peng L., Dai Z., Yin X., Liu R., Li Y. (2024). Association between triglyceride-glucose related indices and mortality among individuals with non-alcoholic fatty liver disease or metabolic dysfunction-associated steatotic liver disease. Cardiovasc. Diabetol..

[27] Chen Z., Qiu X., Wang Q., Wu J., Li M., Niu W. (2024). Serum vitamin D and obesity among US adolescents, NHANES 2011-2018. Front. Pediatr..

[28] Burgevin M., Lacroix A., Ollivier F., Bourdet K., Coutant R., Donadille B., Faivre L., Manouvrier-Hanu S., Petit F., Thauvin-Robinet C. (2023). Executive functioning in adolescents and adults with Silver-Russell syndrome. PLoS ONE.

